# Barriers and facilitators to implementing person-centred dementia care in long-term care facilities in Western and Asian countries: a scoping review

**DOI:** 10.3389/fpsyt.2024.1523501

**Published:** 2025-01-14

**Authors:** Xin Guan, A-min Duan, Gong-kai Xin, Jan Oyebode, Yu Liu

**Affiliations:** ^1^ School of Nursing, China Medical University, Shenyang, Liaoning, China; ^2^ School of Nursing, Li Ka Shing (LKS) Faculty of Medicine, The University of Hong Kong, Pokfulam, Hong Kong SAR, China; ^3^ Centre for Applied Dementia Studies, University of Bradford, Birmingham, United Kingdom

**Keywords:** staff, dementia, person-centred care, barriers, facilitators, long-term care, scoping review

## Abstract

**Background:**

There is a gap between the principles of person-centred dementia care and their actual implementation. However, scoping reviews of the barriers and facilitators to implementing person-centred dementia care in long-term care facilities for Western countries and Asian countries are lacking.

**Objective:**

To identify and compare the barriers and facilitators to implementing person-centred dementia care in long-term care facilities between Western and Asian countries.

**Methods:**

In line with Arksey and O’Malley’s methodology, a scoping review was conducted and is reported following PRISMA-ScR guidelines. Nine English language databases and three Chinese databases were searched to identify qualitative and quantitative research studies published in English and Chinese. Thematic analysis was used to summarise and characterize the barriers and facilitators to implementing person-centred dementia care in long-term care facilities for Western and Asian countries.

**Results:**

Thirty-three studies were included. Over half were conducted in Western countries (n =20). Barriers and facilitators were grouped under four high level themes: Nursing and care staff factors, people living with dementia and family factors, organizational factors, and resource factors. Inadequate knowledge of person-centred care, staffing shortages, time constraints, and low wages were the principal barriers to implementing person-centred dementia care in both Western and Asian countries.

**Conclusions:**

The findings indicate that staff encounter numerous obstacles and needs in implementing person-centred care for people living with dementia in long-term care settings. Educational levels of nursing staff in Western countries were generally higher compared to Asian countries. Additionally, work-related injuries and stigma associated with dementia care presented unique challenges for nursing staff in Asia and were not cited in Western studies. Conversely, family-related factors were more frequently and elaborately cited as influencing person-centred dementia care in Western long-term care facilities. Moreover, Asian studies identified a significant lack of educational training support for person-centred dementia care, as well as shortages in staffing and poor availability of personalized, home-like environments

## 1 Introduction

Person-centred dementia care (PCDC) is characterized by an approach that is individualized and based on holistic understanding of how dementia affects each person in the context not only of their cognitive difficulties but also their personality, biography, health and relationships (1). The terms PCDC and person-centred care are often used synonymously but person-centred care is also used in a wider sense to refer to any individualized care (2). Therefore in this paper, we used the term PCDC throughout, to refer to care based on Kitwood’s model for understanding each individual person with dementia. The person-centred approach to dementia care was developed more than 25 years ago (3), and is now considered the gold-standard practice in care (4). Alzheimer’s Disease International (2022) recommend that care should be person-centred as well as culturally appropriate. Despite this wide recognition of its value, a gap between theory and practice persists, with significant disparities in PCDC across different countries and regions. For instance, it has been found that in China the prevailing approach to dementia care is often characterized by a medical focus, a disease-centric perspective, and a task-oriented methodology (5, 6). Care providers typically emphasize routines and tasks over the personalized preferences of individuals with dementia (7). In contrast, Western Europe, North America and Australia have seen a shift in the last twenty years towards the aspiration to deliver PCC that is relationship-driven, collaborative, and holistic. This approach prioritizes the quality of life of people with dementia by fostering a sense of community and belonging between staff and care recipients (8).

Due to the earlier demographic transition in Western countries (9), which led to an ageing population, strategies such as PCDC were initially developed in these regions to address the increasing number of people developing dementia (10). Asian countries have more recently undergone demographic changes, and have adopted best practices in dementia care, including PCDC, from Western countries to guide their developing dementia services as their populations age (11). PCDC is founded on caring by understanding each person’s needs. Expression of individual needs and viewpoints is consistent with the Western ethos of individualism and freedom (12), which may facilitate the implementation of PCDC. By contrast, the influence of Confucianism in Asian countries, means these cultures often emphasize collective interests, with individual needs frequently being subsumed under the broader community agenda (13). Asian cultures value humility, leading to more indirect and restrained personal expression. This cultural backdrop may mean those with dementia feel uncomfortable expressing their needs and may also subtly influence the attention long-term care staff pay to unique individual needs (14). These could be barriers to the adoption of PCDC. Thus, questions have been raised about the effectiveness of transferring care principles across different cultural and systemic contexts (15). Comparing PDCD across these two contexts can help to understand whether the concept is transferable between environments, address its applicability in these new settings and identify whether it might need adaptation in its implementation (16). Consequently, this paper aims to compare key differences and similarities in barriers and facilitators to PCDC between Western and Asian countries. This should have significant practical implications and provide theoretical and empirical insights.

Several reviews have addressed the barriers and facilities to implementing PCDC experienced by nursing home staff (17–19). However, to date there have been no comprehensive scoping reviews comparing the barriers and facilitators of PCDC between Western and Asian countries. Kim and Park’s (18) review excluded observational and qualitative studies, while Guney et al. (17) restricted their review to qualitative studies of the perceptions of nurses and nursing assistants. The review by Kim and Park includes studies exclusively from Western countries, while Güney’s review contains only one study from an Asian country (South Korea), with the rest being from Western countries. In addition, Lee et al. (19) was limited to qualitative studies reporting the experience of implementation of PCDC, and the included countries were mainly Western, so excluding Asian literature. Conversely, Wang et al. (6)’s meta-synthesis only reports on PCDC in China. Searches of the Cochrane Library, PROSPERO, and JBI Library of Systematic Reviews confirmed that similar reviews did not exist.

To facilitate the implementation of PCDC, it is essential to understand the contextual barriers to providing this type of care, especially as some of these may be culturally determined or influenced by the societal context. In this review, we have defined Western countries as those with cultural ties to Europe, including nations in Western Europe, North America, and Australasia, such as the U.S., Canada, Australia, and New Zealand (20). These countries have the longest established aging populations. Asian countries are defined as those located on the Asian continent, including East Asia (e.g., China, Japan), Southeast Asia (e.g., Thailand, Vietnam), South Asia (e.g., India, Pakistan), Central Asia (e.g., Kazakhstan), and Western Asia (e.g., Saudi Arabia, Turkey) (21). These countries generally share a more collectivist culture and have experienced rapid ageing of their populations more recently. Barriers refer to factors that hinder the implementation of PCDC in long-term care facilities, and facilitators refer to factors that promote the implementation of PCDC. Therefore, in this review, we compare facilitators of and barriers to the implementation of PCDC between Asian and Western countries for people with dementia who are receiving long-term care.

## 2 Methods

In accordance with the methodology of Arksey and O’Malley (22), we conducted a scoping review. We report our study in accordance with the Preferred Reporting Items for Systematic Reviews and Meta-Analyses statement (23) for reporting scoping reviews. Through several meetings, the review team developed the protocol for conduct of each stage of the review. As this paper aims to provide a comprehensive overview rather than evaluate the quality of the literature, in line with Arkey and O’Malley’s approach, a quality assessment was not conducted (22).

### 2.1 Research question

Our purpose was to synthesize research literature on facilitators of and barriers to the implementation of PCDC in long-term care facilities in Asian and Western countries. We asked two main research questions:

1. What are the facilitators of and barriers to the implementation of PCDC in long-term care facilities in Asian and Western countries?

2. How do the facilitators of and barriers to the implementation of PCDC in long-term care facilities compare between Asian and Western countries?

### 2.2 Search strategy

The phenomena of interest were identified, a research context was established, and a framework for the search terms was created (24). The phenomena of interest included facilitators of and barriers to the implementation of PCDC. The research context was long-term care facilities in Asian and Western countries. Formal caregivers play a pivotal role in providing PCDC and face unique challenges critical to improving care quality. Therefore, our study included participants who were formal caregivers, either nursing or other paid staff. Where studies also included people living with dementia and their family members, this was acceptable as long as formal caregivers were included. Our focus was on the barriers and facilitators experience by formal caregivers. These could be at an individual level or within the system of care, such as organizational, cultural, social, political, and economic influences on delivery of PCDC (Specific inclusion and exclusion criteria are provided in (**Table 1**).

**Table 1:** Inclusion and exclusion criteria.

	Inclusion criteria	Exclusion criteria
Participants	Had to include nursing staff (e.g., nurses, nurse aides, caregivers)	
Phenomena of interest	Studies explored barriers and facilitators to person-centred care for persons living with dementia, with or without implementation	
Context	Long-term care settings (e.g., nursing home, long-term care facility, residential home, care home, assisted living, residential aged care)	Non-Asian or Non-European countries
Design and publication type	Any type of original research	Any publication not available in English or Chinese. Publications not available in full text (e.g., editorial, abstracts, commentary, or thesis)

We consulted an information science expert and a research librarian to develop suitable search strategies. Using nine international databases (Cochrane Library, Cumulative Index to Nursing and Allied Health Literature, Embase, Medline, PsycINFO, ProQuest, PubMed, Scopus, and Web of Science) and three Chinese databases (China National Knowledge Infrastructure (CNKI), Wanfang, and China Biology Medicine (CBM)), we conducted a comprehensive search. The search period began with database inception and ended in November 2024. We restricted language to Chinese and English. The search strategy for Chinese-language studies used Chinese search terms and was based on CNKI, and the search strategy for English-language studies used English terms and was based on PubMed (see **Table 2**). Using mesh subject headings (MESH) and free-text keywords for “nurse” or “nursing,” we then modified the strategy to be suitable for each database (see the Supplementary Material). We did not restrict search terms on study design; however, we did not hand search journals or supplementary grey materials. All the studies included in our scoping review are provided in the reference list.

**Table 2:** Steps and detailed search terms used in PubMed and CNKI.

Database	Step	Search terms
PubMed	1	Subject area P1:dementia (Dementia[MeSH Terms]) OR (Alzheimer Disease[MeSH Terms])) OR (Dementia*[Title/Abstract])) OR (alzheimer*[Title/Abstract])
	2	Subject area P2:Long-term care (Long-Term Care[MeSH Terms]) OR ("nursing home*"[Title/Abstract]) OR ("residential home*"[Title/Abstract]) OR ("care home*"[Title/Abstract]) OR ("assisted living"[Title/Abstract]) OR ("residential aged care"[Title/Abstract]) OR (longterm care[Title/Abstract])
	3	Subject area I: person-centered care (Patient-Centered Care[MeSH Terms]) OR ("person centered care"[Title/Abstract])) OR (“Patient centred”[Title/Abstract])) OR (“Person centred”[Title/Abstract])) OR ("person centered dementia care"[Title/Abstract])) OR ("Patient Centered Nursing"[Title/Abstract])) OR ("person centered care"[Title/Abstract])) OR ("person centred care"[Title/Abstract])) OR ("patient centred care"[Title/Abstract])) OR ("person centered dementia care"[Title/Abstract])) OR ("person centred dementia care"[Title/Abstract])) OR ("Patient Centered Nursing"[Title/Abstract])
	4	Subject area O:barriers and facilitators (Perspective*[Title/Abstract]) OR (view*[Title/Abstract])) OR (attitude*[Title/Abstract])) OR (Opinion*[Title/Abstract])) OR (Understanding[Title/Abstract])) OR (experience*[Title/Abstract])) OR (meaning[Title/Abstract])) OR (Belief*[Title/Abstract])) OR (Customs[Title/Abstract])) OR ("cross cultur*"[Title/Abstract])) OR (cultur*[Title/Abstract])) OR (ethnic*[Title/Abstract])) OR (Barrier*[Title/Abstract])) OR (Facilitator*[Title/Abstract])
CNKI	1	Theme = Person-Centered + People-Oriented
	2	Theme = Alzheimer's + Dementia + Cognitive Impairment

### 2.3 Eligibility and study selection

Title and abstract screening, in accordance with our inclusion criteria, as well as full-text review, were conducted by GX and Am-D to ensure consistency and confirm the inclusion of relevant articles. To extract data from the included studies and to synthesize evidence, we used a standardised, piloted form. Authors, country, year, title, objectives, study design and setting, participants, data collection, type of analysis, and main findings or conclusions were extracted and tabulated. We also extracted the facilitators of and barriers to the implementation of PCDC in long-term care facilities in Asian and Western countries. After data extraction by one review author (XG), other members of the review team (Am-D, YL, JO) cross-checked the findings. The review team met regularly to discuss and resolve any ambiguity.

### 2.4 Collating the results

Braun’s thematic analysis method (25) was used to collate the findings into themes and categories of facilitators and barriers to dementia care. The process involved several steps: Firstly, the included literature was thoroughly read to gain familiarity with its content. Secondly, the material on barriers and facilitators to PCDC that had been extracted was coded into initial themes. We assigned initial codes to qualitative study findings and to variables from quantitative studies. Thirdly, initial codes were categorized into latent themes and sub-themes. Fourthly, potential themes and sub-themes underwent further scrutiny and validation, based on criteria of internal homogeneity and external heterogeneity. Some themes were subsequently divided, merged, or removed. Finally, each theme was clearly defined and labelled.

Coding, thematic categorization, and theme refinement were conducted independently by two researchers, with subsequent discussions to ensure consistency and accuracy (XG, Am-D). In cases of disagreement, consensus was reached through discussion with two experienced qualitative research professors (JO, YL) to ensure the best possible categorisation. Excel software facilitated organization, coding, and validation of the data analysis process.

## 3 Results

### 3.1 Study selection

**Figure 1:**
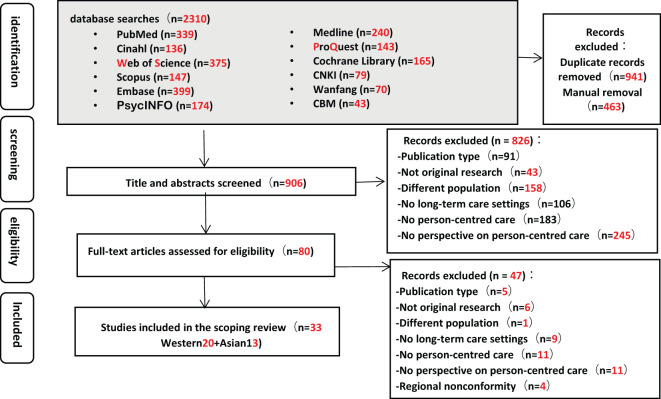
PRISMA diagram.

Of the 2,310 articles identified in the search, 80 underwent full-text screening, resulting in final selection of 33 studies (5, 6, 26–56) (see **Figure 1**). The steps followed and the number of records included or excluded at each stage are summarised in **Figure 1**.

### 3.2 Characteristics of included studies

The characteristics of the included studies are listed in **Table 3**. Most studies (n=24) (5, 6, 26–37, 39, 41, 43, 45, 47, 50, 53–56) were published after 2020, with the publication period ranging from 2014 to 2023. Only one Asian paper had been published pre-2020, whereas eight Western papers had been published pre-2020. More than half (n=20) (26, 28, 39–56) were conducted in Western countries, including the Netherlands (n=4) (53–56), the United States (n=3) (50–52), Australia (n=3) (47–49), Sweden (n=3) (26, 28, 41), Canada (n=2) (45, 46), Ireland (n=2) (43, 44), and others (n=3) (39, 40, 42). Twelve studies were conducted in Asian countries, including China (n=6) (5, 6, 35–38), South Korea (n=5) (27, 31–34) and others (n=2) (29, 30). One of the studies (56) used mixed methods, nine (31, 34, 37, 38, 40, 41, 53–55) used quantitative methods and twenty-three (5, 6, 26–30, 32, 33, 35, 36, 39, 42–52) employed a qualitative design (see **Table 3**). Across all studies, a total of 4,879 care staff participated, with sample sizes ranging from 7 to 1,161 participants. Qualified nurses and nursing assistants constituted 95% of the participants, while the remainder were other healthcare professionals, individuals with dementia and family carers. Twenty-nine studies (5, 6, 26, 27, 30–41, 43–52, 54–56) included qualified and assistant nurses. Other studies also included managers (26, 39, 41, 42, 49, 50), welfare staff (54), healthcare professionals (29), and substitute decision-makers (45). Doyle and Rubinstein (52) observed people with dementia as well as formal caregivers, while three studies (43, 47, 49) interviewed care staff, people living with dementia, and family members. The majority of the research was conducted in nursing homes (n=14) (26–28, 30, 32, 35, 39–41, 44, 50, 51, 55, 56). Other care facilities were variously described as residential care (53), long-term care homes (6, 31, 33, 34, 36, 43, 45, 46, 49, 53), dementia-specific long-term care (52), residential aged care (37, 48), aged care homes (47) and long-term-care hospitals (31, 34).

**Table 3:** Study characteristics and main findings of included studies (N=33).

Author, year, country	Title	Aims	Design	Setting	Participants	Data collection and analysis	Main findings and/or conclusion
Kloos et al. (2020), Netherlands (56)	Exploring facilitators and barriers to using a person centered care intervention in a nursing home setting	To investigate perceived facilitators and barriers to use and implementation of a PCC intervention using the Implementation Framework of Innovations in the healthcare setting	Explorative mixed method study	NHs (n=17)	Nursing staff. Interviews (n=11) with a longitudinal survey (n=132), actual implementation 3 months later (n= 63)	Chi square and logistic regression. the core elements of the MIDI determinant list	Perceived barriers and facilitators depended on the intervention components. Assessment of resident well-being required a stable nursing home context and a detailed implementation plan. Planning of well-being support was impeded by lack of knowledge. Behavioral changes in nursing care required easy integration in daily caring tasks and social support.
Rutten et al. (2021), Netherlands (55)	Work environment and person-centred dementia care in nursing homes-A cross-sectional study	To explore the relationship between work environment, job characteristics and PCC for people with dementia in NHs.	Cross-sectional quantitative study	NHs (n = 49)	Direct care staff (n=552)	Multilevel linear regression analyses	Leaders may consider facilitating collaboration and creating unity between care staff, clients and family members to provide PCC. Transformational leadership, educational programmes and leadership coaching are recommended.
Boumans et al. (2021),Netherlands (54)	How staff characteristics influence residential care facility staff’s attitude toward person-centered care and informal care	To explore association between staff characteristics (age, education, years of work experience, function - care or welfare) and staff attitudes toward PCC provision and including informal caregivers in the caregiving process in residential care facilities.	Cross-sectional quantitative study	Residential care facilities (n=2)	Care staff (nurses, nurse assistants) (n=68) welfare staff (activity counselors, hostesses, living room caretakers)	Shapiro-Wilk test; Multiple linear regressions	Higher age of care and welfare staff was associated with more negative attitude toward PCC and informal care provision. Welfare staff had less positive attitudes toward informal care provision. Future observational and interview studies should collect evidence on reasons for negative attitudes of older staff members, to be able to target interventions that eliminate or reduce these negative attitudes.
Kunz et al (2022)., Netherlands	From dementia mindsets to emotions and behaviors: Predicting person-centered care in care professional	To better understand facilitators of PCC, focusing on individual characteristics of care professionals	Cross-sectional study	Long-term care facilities (n=1)	Care professionals (n=370)	Directed content analysis	The study extends knowledge of facilitators (positive emotional responses to care situations) and barriers (fixed dementia mindset) to PCC in dementia care professionals. Dementia mindsets and emotional responses to care situations may be a fruitful target for training care professionals.
Doyle et al. (2014),USA (52)	Person-Centered Dementia Care and the Cultural Matrix of Othering	To examine how PCC was defined, shaped, and practiced by staff members within a dementia care setting	Ethnographi-c research	Dementia-specific long-term care setting	People with dementia (n=20) and staff members (n=25)	Ethnographic approach	Three main characteristics (dementia as a master status, functional dependence, aggressiveness) that could reduce the extent of othering and improve person centredness of elder care settings.
Kolanowski et al. (2015),USA (51)	"Wish we would have known that!" Communication Breakdown Impedes Person-Centered Care	To understand how NH staff obtain information needed for implementing PCC to residents with dementia who exhibit BPSD, and how they communicate this information to other staff. Barriers to PCC and information exchange were also explored.	Focus group methodology	NHs (n=2)	Staff (n=59)	Qualitative content analysis	To improve PCC information exchange requires: inclusion of all staff, particularly CNAs; communication systems that consider time and resource constraints of NHs; development of educational programs for BPSD that are responsive to staff learning styles; administrative investment in nursing leadership; and reimbursement approaches to encourage culture change investments.
Bhattacharyya et al. (2022),USA (50)	Person-Centered Care in Nursing Homes: Potential of Complementary and Alternative Approaches and Their Challenges	To examine barriers and facilitators to implementation of PCC and how the integration of complementary and alternative approaches has potential to improve residents’ quality of life in NHs	Focus group	NHs (n=5)	Managers (n=36) and direct care or auxiliary workers (n=44)	A qualitative descriptive approach	NHs offer many engagement activities, but these are not purposefully integrated into a PCC plan. Turnover, “working short,” supervisor support, and rising resident care needs make it challenging to implement PCC in NHs. This knowledge can help identify and improve strategies for providing deeper, more meaningful support. Complementary and alternative approaches have potential to be therapeutic if integrated into collaborative approaches to care.
Chenoweth et al. (2015), Australia (49)	PerCEN trial participant perspectives on the implementation and outcomes of person-centered dementia care and environments	To understand managers, nurses, care staff, families, and PCC/PCE facilitators’ views on: (i)inconsistencies in PerCEN study findings (ii) whether PCC and PCE impacted on quality of care services and outcomes (iii) factors that enabled and inhibited PCC and PCE implementation.	A qualitative study	Long-term care homes	Care managers (n=29), nurses and care staff (n=70); telephone surveys with family members (n=73)	Content analysis, code building, theme development, and synthesis of findings	Successful knowledge translation of the PCC model starts with managerial leadership and support; it is sustained when staff are educated and assisted to apply the model, and, along with families, come to appreciate the benefits of flexible care services and teamwork in achieving resident well-being.
Oppert et al. (2018), Australia (48)	Knowledge, facilitators and barriers to the practice of person-centred care in aged care workers: a qualitative study	To reveal the level of understanding of ACWs of PCC principles, and barriers that prevent the practice of PCC.	A qualitative study	Residential aged care facilities (n=2)	Direct carers (n=12)	Thematic analysis	Aged care workers have a reasonable but incomplete understanding of PCC. Insufficient time and residents’ dementia behaviours were barriers to care workers’ provision of PCC. Teamwork facilitated PCC by increasing instrumental and relationship resources
Seah et al. (2022), Australia (47)	Person-centred Australian residential aged care services: how well do actions match the claims?	To investigate perspectives of a convenience sample of older residents, family members and staff on whether services that claimed to be person-centred were person-centred.	A qualitative study	Seven aged care homes	Residents (n=12), family members (n=15) and staff members (n=18)	Deductive thematic analysis using VIPS framework	Ten themes reflected failings in implementing PCC in 5/7 homes and four themes reflected adherence to PCC in 2/7 homes. Few homes fully appreciated the requirements of a system-wide person-centred aged care service.
Lindner et al. (2023), Sweden (41)	Person-centred care in nursing homes during the COVID-19 pandemic: a cross sectional study based on nursing staff and first-line managers’ self-reported outcomes	To evaluate nursing staff and manager perceptions of the opportunities to perform PCC during the COVID-19 pandemic	A cross- sectional study	NHs (n=9) and one short stay residence	Nursing staff (n=463) and First Line Managers (FLM) (n=8)	Kruskal-Wallis test and multiple regression	Despite the COVID-19 restrictions and the criticism directed against care of older people; the day staff felt that they conducted PCC. Staff in NHs for dementia had the highest opportunities for PCC – possibly because they are better prepared to provide care. The importance of leadership was evident, which means that investment in FLMs is seen as necessary.
Annica et al. (2023), Sweden	Nursing home managers' descriptions of multi-level barriers to leading person-centred care: A content analysis	To explore barriers to leading PCC as narrated by nursing home managers.	A descriptive qualitative design	NHs (n=3)	Nurses (n=9)	Qualitative content analysis	The integration of salutogenesis and Sense of Coherence into PCC enhanced residential aged care by highlighting the resources of older persons, leading to a more holistic approach. Additionally, this method improved work satisfaction among care personnel.
Sofia et al. (2024), Sweden	Nurses’ experiences of integrating the salutogenic perspective with person-centered care for older people in Swedish nursing home care: an interview-based qualitative study	To describe nurses’ experiences of combining PCC with a salutogenic approach at a NH for older people	Semi-structured interview	NHs (n=11)	NH managers (n=12)	Qualitative content analysis	Barriers to implementing PCC were identified at various levels. On the personal level, prioritizing professional and family considerations over resident needs was a significant barrier. At the team level, differences in care values, processes, and priorities, along with staff turnover and low foundational knowledge, posed challenges. Organizationally, financial constraints, functional building design, and group-level rostering were notable obstacles.
Hunter et al. (2016),Canada (46)	A qualitative study of nursing assistants' awareness of person-centred approaches to dementia care	To improve educational programming by identifying potential gaps in PCC practice of NAs and to debate potential of employee education by evaluating whether gaps in best practice dementia care present a problem in knowledge.	A qualitative study	LTC homes (n=7)	NAs Nursing assistants (n=7)	Qualitative content analysis	Application of PCC strategies varies across NAs. The authors propose ways of enhancing NA education in order to address gaps in knowledge and recommend sustained attention to organisational factors that contribute to variability in practice.
Charlene et al. (2021),Canada	Perspectives of substitute decision‐makers and staff about person‐centred physical activity in long‐term care	To explore the care processes that best exemplify PCC during physical activity (PA) for LTC residents with dementia from the perspectives of substitute decision‐makers (SDMs) and LTC home staff.	Semi-structured interviews	LTC homes (n=2)	Substitute decision-makers SDM (n = 26) and staff (n = 21)	Thematic content analysis	Understanding the care processes that are most recognized as PCC and valued by SDMs and LTC home staff has implications for education and training. Insights into SDMs' care expectations regarding PCC can inform staff about which actions should be prioritized to meet care expectations and can foster relationships to the benefit of residents with dementia.
Colomer et al. (2016),Ireland (44)	Person-centred dementia care: a reality check in two nursing homes in Ireland	To identify perspectives and experiences of care assistants with PCC in the NH in which they worked. Specific objectives included to address: . knowledge, education and attitudes around PCC, . obstacles and challenges around the implementation of PCC.	Semi-structured interviews	NHs (n=2)	Care assistants (n=13)	A phenomenological approach	Considerable disparity between policy and practice, in particular because care assistants lacked clarity on what PCC is and reported that they were not educated in it. Besides the necessity of more (and more explicit) training on PCC, the findings also suggested concerns around communication between staff and management and the need for improvement of staffing resources and available time in residential settings in order to make the delivery of PCDC a reality.
Hennelly et al. (2022) Ireland (44)	A multiple perspective view of personhood in dementia	To explore the interpretation and application of personhood within formal care provision for people with dementia in Ireland.	A multiple perspective study design	Community and long-term care settings.	People with dementia (n=8), family carers (n=8) and formal carers (n=15)	Phenomenological analysis	General consensus on the core elements of personhood among all participants: interests and preferences; lifecourse experiences; social interaction; family; and place. However, there was ambiguity among family caregivers and formal caregivers in the interpretation of changes to personhood as the disease progresses. Interpersonal and structural barriers to supporting personhood were identified by all participants.
Stacpoole et al. (2017),UK	Implementing the Namaste Care Program for residents with advanced dementia: exploring the perceptions of families and staff in UK care homes	To establish whether the Namaste Care program can be implemented in UK care homes; and what effect Namaste Care has on the quality of life of residents with advanced dementia, their families and staff.	An organisational action research methodology	Care homes (n=6)	Care home managers (n=9)	A qualitative descriptive approach	Namaste Care can enrich the quality of life of older people with advanced dementia in care homes. It was valued for the benefits seen in residents; the improvement in relationships; and the shift towards a person-centred, relationship-based culture of care. Namaste Care deserves further exploration and investigation including a randomised controlled trial.
Røen et al. (2018),Norway (40)	Person-centered care in Norwegian nursing homes and its relation to organizational factors and staff characteristics: a cross-sectional survey	To explore and understand the association between PCC and organizational, staff and unit characteristics in NHs (NHs).	Cross-sectional study	NHs (n=175)	Staff (n=1161)	Multilevel linear regression modelling	Shows an association between PCC and organizational, staff and unit characteristics in NH. Providing PCC in NH care is closely linked to how staff experience their job situation in addition to organizational and structural factors and the physical environment. Attention needs to be given to such factors when planning NH care.
Richter et al. (2022),Germany (39)	Factors influencing the implementation of person-centred care in nursing homes by practice development champions: a qualitative process evaluation of a cluster-randomised controlled trial (EPCentCare) using Normalization Process Theory	To identify facilitators and barriers of the implementation of PCC in German NHs from the perspective of participating practice development champions.	A qualitative design	NHs (n=18)	Staff and managers (n=66)	Inductive coding	Facilitating implementation factors included broadening of the care perspective (coherence), tolerance development within the care team regarding challenging behaviour (cognitive participation), testing new approaches to solutions as a multi-professional team (collective action), and perception of effects of PCC measures (reflexive monitoring). Facilitating factors reported in all the NPT constructs, affecting the entire implementation process, were the involvement of relatives, multi-professional teamwork and effective collaboration with physicians.
MaoPan et al. (2016),China	Investigation on the current situation of person-centered dementia care in elder care institutions in Huna province	To investigate the status of PCC in dementia care units in Hunan Province and exploring its influencing factors to improve PCC services	A cross-sectional survey	Aged care facilities (n=20) in HuNan Province	Formal caregivers (n=112)	Multi-stage cluster random sampling	The age and education level of caregivers were the influencing factors for the level of PCC services in dementia care units of elderly institutions. The level of PCC in dementia care units in Hunan Province needs to be improved, and the age structure of caregivers should be further optimized and the education level of caregivers should be improved, to improve the level of PCC.
Dai, Yunyun et al. (2020),China (37)	Caregivers' Dementia Knowledge and Care Approach in Residential Aged Care Facilities in China	To investigate the dementia knowledge and care approach used by caregivers in residential aged care facilities in China.	A cross-sectional survey	Residential aged care facilities in China. (n=34)	Formal caregivers(n=785)	A stratified cluster sampling process Descriptive analyses	The majority of caregivers showed limited knowledge of dementia and tended not to adopt a person-centred approach to care. Educational level, dementia care training, and years of work experience were positively associated with dementia knowledge. Educational level and years of work experience were also associated with a person-centred approach to care.
Zhao, Yayi et al. (2020),China	Understanding dementia care in care home setting in China: An exploratory qualitative study	To explore the current dementia care practices in care home setting in China because people with dementia have increased need for residential care as the cognitive function worsens.	An exploratory qualitative study	Care homes (n=4) in a metropolitan city in China	Staff members (n=15) working at the care home	Qualitative content analysis	Four categories about dementia care practices in care homes were identified: (a) care environment (hospital-like layout, inappropriate lighting, environmental noise, inappropriate use of colour and unclear signage), (b) care culture (being medical-oriented, overlooking individual uniqueness and privacy), (c) attitudes towards dementia (treating as children, being authoritative, adopting punitive approaches, trying to respect the residents and having a positive learning attitude) (d) dementia care competence (questing for specific training and resources, questing for culturally specific practices and strengthening communication with family).
Junqiao Wang et al. (2022),China	Understanding person-centered dementia care from the perspectives of frontline staff: Challenges, opportunities, and implications for countries with limited long-term care resources	To explore how caregivers who provide care for people with dementia understand PCC in their daily work.	Qualitative research	Non-profit LTC facilities with dementia care units in Shanghai (n=5)	Frontline staffs/nursing care aides (n=40)	Purposive sampling and conventional content analysis	①Infantilizing older residents with dementia and labeling them using wisdom gained through experience as a mother can prevent meaningful, equal, and person-centred conversations. ②Care aides do not have regular formal interactions and sensemaking with nurses and other professionals in NHs. ③Increasing interactions and communication between care aides and health care professionals in NHs can lead to insight for changing the approach to in-service training to achieve better acceptance by care aides.
Zhao, Yajie et al. (2022),China	Facilitators and barriers to implementing person-centered care among patients with dementia in long-term care facilities	To explore facilitators and barriers to implementing PCC among residents with moderate to severe dementia in long-term care facilities.	Descriptive qualitative research	Long-term care facilities (n=2)	Formal caregivers(n=11)	Purposive sampling Content analysis	Facilitating factors included the care plan, the organization and implementation and external support. Hindering factors come from the person with dementia and the organization and implementation process. The use of multidisciplinary team discussions to determine a flexible care plan that matches the person with dementia and the arrangement of caregivers who are familiar with the person with dementia to guide them to participate in group activities, and the joint support of the hospital district, caregivers, and family members can facilitate PCC.
Zhou Yiping et al. (2023),China	A qualitative research on the experience of the senior nursing staffs for dementia care	To deeply understand the experience of senior nursing staffs for dementia care in China and provide a basis for the targeted intervention implementation of dementia care.	Qualitative research	NH (n=1)	Senior nursing staffs (n=7)	Colaizzi's seven-step method of data analysis	Nursing staff experience is multi-dimensional, with a heavy care burden and great psychological pressure but nursing staff have a sense of person-centred belief and good communication literacy. The results may provide a reference for the implementation of a dementia care competency intervention and training course.
Chang, HeeKyung et al. (2020), Korea (34)	Person-Centered Care, Job Stress, and Quality of Life Among Long-Term Care Nursing Staff	To investigate correlations among job stress, quality of life, and PCC of nurses as well as factors affecting the person-centred care abilities of nursing staff working at long-term care hospitals.	A cross-sectional survey	Long-term care hospitals (n=3) in South Korea	Nursing staff (n= 183)	Cross-sectional study descriptive analysis and regression modelling.	Significant factors associated with PCC included quality of life and job stress of the participants. The regression model with job stress and quality of life as predictor variables accounted for 29.2% of the variance in PCC. Higher quality of life and lower job stress were increased PCC abilities of nurses in long-term care facilities.
JiSun CHoi et al. (2020), Korea	Person-Centered Care Environment Associated With Care Staff Outcomes in Long-Term Care Facilities	To examine the relationship between PCC environments and staff outcomes, including job satisfaction and turnover intention, among care staff in Korean long-term care facilities	Descriptive, correlational study	Long-term care facilities in Korea (n=13)	235 care staff (n=235) comprising nursing staff (n=94) and personal care workers (n=141)	Purposive sampling Descriptive statistics	After controlling for individual (age, education, monthly income, position, shift work, job tenure) and organizational (type of facility, location, ownership, bed size, staffing levels) characteristics, a significant relationship was found between the PCC environment and job satisfaction and turnover intention among staff in Korean long-term care facilities.
Eun-Hi Kong et al. (2021),Korea	Nursing home staff's perceptions of barriers and needs in implementing person-centred care for people living with dementia: A qualitative study	To explore NH staff's barriers and needs in implementing PCC for people with dementia.	A qualitative study	NHs (n=6) in Korea.	Staff members (nurses, nurse's aides, or care workers) (n=24)	Convenience sampling method. Qualitative content analysis	Four themes from data analysis: insufficient resources, lack of education, negative mindset, poor relationships. NH staff experienced many barriers and unmet needs in implementing PCC for people with dementia
Park et al. (2021) Korea	A predictive model of the perceptions of patient-centered care among nurses in long-term care hospitals: A cross-sectional study	To propose and examine a predictive model of the impacts of organizational and individual factors on the perceptions of PCC among nurses working in long-term care hospitals.	A cross-sectional study	Long-term-care hospitals in South Korea (n=6)	Nurses (n=187)	Descriptive statistics.	The model explained the impacts of the factors on how nurses perceive PCC, it explaining 47% of the variation in PCC. Organizational factors had stronger influences on PCC [innovative organizational culture, teamwork compared to individual factors.
Dayeong et al. (2024) Korea	Nurses' Shared Subjectivity on PersonCentered Care for Behavioral and Psychological Symptoms of Dementia in Nursing Homes	To explore and gain insight into the shared subjective perspectives of nurses on providing PCC to manage BPSD in NHs in order to elicit a deeper understanding of how nurses interpret and approach the provision of PCC	Q methodology	NHs (n=5)	Nurses (n=30)	An inverted factor analysis	Four key factors from nurses' perspectives on person-centered care were identified: (a) sharing detailed information to update care strategies, (b) monitoring to identify residents' true needs, (c) being aware of interactive cues in relationships, and (d) linking an individual's life pattern to their current care.
Strøm et al. (2021), India (30)	Nursing Staff's Knowledge and Attitudes towards Dementia in an Indian Nursing Home: A Qualitative Interview Study	To explore the knowledge about and attitude towards dementia among nursing staff working in residential care facilities for older people in India	An explorative and descriptive qualitative design	NHs (n=6)	Nurses (n=8) and care assistants (n=4)	Purposive sampling content analysis	Highlighted 3 dimensions in relation to staff knowledge of and attitudes toward dementia in residential care facilities in India: (1) people with dementia – a walking mystery, (2) we need to go along with them, but it is challenging, and (3) if we know, we can care for them in a better way.
Shrestha et al. (2022),Nepal (29)	Dementia care in Nepalese old age homes: Critical challenges as perceived by healthcare professional	To explore and describe critical challenges in current dementia care practice as perceived by healthcare professionals (HCPs) in old age homes (OAHs) in Kathmandu, Nepal.	An exploratory qualitative design	Old age homes (OAHs)(n=5)	Healthcare professionals (HCPs) (n=11)	Purposive sampling in-depth interviews	Limited educational training, sparse competence in mastering residents’ cognitive disturbances and BPSD, insufficient resources to ensure sufficient numbers of HCPs and MDs for proper diagnostic examination, treatment and dementia specific care were identified as critical challenges restricting quality dementia care in these Nepalese OAHs.

PCC, person-centred care; MIDI, Measurement Instrument for Determinants of Innovations; BPSD, behavioral and psychological symptoms of dementia; CNAs, certified nursing assistants; NHs,nursing homes; PerCEN, Person-Centred Dementia Care and Environment; VIPS, Social Environment’ framework; NH, nursing homes; NPT, Normalization Process Theory; LTC, long‐term care.

### 3.3 Key findings

The studies included indicated that nursing staff experienced multiple facilitators of and barriers to the implementation of PCDC for people with dementia in long-term care facilities. Across the 33 studies, we identified 67 facilitators and 122 barriers to the implementation of PCC. In both Asian and Western countries, the facilitators of and barriers to care could be grouped into four broad themes. These were factors associated with people living with dementia and their families, with nursing and care staff, with resources, and with organizational influences. Descriptions of each theme and the associated barriers and facilitators are presented in **Table 4**.

**Table 4:** Barriers and facilitators to implementing person-centred care in long-term care facilities. (Number of studies shown in brackets).

Theme	Barriers		Facilitators	
	Western	Asian	Western	Asian
**Nursing and care staff factors**	The characteristics of nursing staff (n=2)	The characteristics of nursing staff (n=3)	Characteristics of the nurses, such as good communication skills (n=1)	Characteristics of the nurses, such as rich working experience and high ability in dementia care (n=3)
	Not understanding patient preferences, forcing patients to accept unwanted care (n=3)	Not understanding patient preferences, forcing patients to accept unwanted care (n=1)	Assess patient preferences, meet patient needs, and respect patients (n=7)	Assess patient preferences, meet patient needs, and respect patients (n=3)
	Negative attitude of nursing staff (n=2)	Negative attitude of nursing staff ( n=1)	Nurses received positive feedback and perceived the importance of PCC (n=2)	Nurses had a positive attitude and were willing to learn and accept the PCC concept (n=2)
	Inappropriate attitude towards people living with dementia, such as labelling them (n=1)	Inappropriate attitude towards people living with dementia, such as treating dementia as a "baby" (n=3)	The nurse empowered the patient and encouraged the person with dementia to exercise maximum independence (n=2)	–
	Difficult to adapt to PCC model (n=1)	Medical-oriented nursing model (n=2)	–	–
	–	Nursing staff experience of injury on the job (n=1)	–	–
	–	Stigma associated with dementia and the work of dementia care (n=2)	–	–
**People living with dementia and family factors**	Lack of family support for work or lack of family involvement, family distrust of nurses, unrealistic family expectations and criticism (n=7)	Lack of family support for work or lack of family involvement, family distrust of nurses, unrealistic family expectations and criticism (n=2)	The family supports the work, or notices the effect (n=4)	The family supports the work, or notices the effect (n=2)
	Disease factors in dementia: physical factors and personality factors (n=3)	Disease factors in dementia: physical factors and personality factors (n=2)	–	–
	Family members lacked PCC knowledge (n=2)	–	–	–
**Organizational factors**	Lack of communication or poor communication (n=3)	Lack of communication or poor communication (n=3)	Effective communication (n=3)	Effective communication(n=3)
	Poor relationships between nurses and caregivers, people living with dementia and their families (n=3)	Poor relationships between nurses and caregivers, people living with dementia and their families (n=1)	Establish good relationship (n=2)	–
	Inadequate cooperation between nursing staff and family members (n=2)	Lack of cooperation (n=2)	Teamwork (n=7)	Teamwork (n=2)
	Inadequate or inappropriate education of PCC for nursing staff and their family members (n=7)	Lack of PCC education (n=5)	PCC training or education (n=3)	PCC training or education (n=4)
	The leader does not support the PCC or leads incorrectly (n=4)	The leader does not support the PCC or leads incorrectly (n=1)	Leadership supports PCC and leads appropriately (n=6)	Leadership supports PCC and leads appropriately (n=2)
	PCC activity set-up issues (n=6)	PCC activity set-up issues (n=1)	The implementation factors of PCC are appropriate (n=3)	The implementation factors of PCC are appropriate (n=1)
**Resource factors**	The physical environment of a long-term care facility does not conform to PCC (n=2)	The physical environment of a long-term care facility does not conform to PCC (n=4)	The warm home feeling of the environment, or the personalized setting of the room (n=2)	The warm home feeling of the environment, or the personalized setting of the room (n=1)
	High turnover of personnel (n=2)	High turnover of personnel(n=2)	–	People with dementia see familiar faces (n=2)
	A lack of staffing (n=11)	A lack of staffing (n=6)	–	–
	A lack of time (n=11)	A lack of time (n=2)	–	–
	Wages are too low (n=3)	Wages are too low (n=4)	–	–

The terms in bold represent the four main themes of the study. They are formatted this way to enhance visibility and draw attention to the key areas of focus within the research.

#### 3.3.1 Nursing and care staff factors

Nursing and care staff’s personal characteristics were important factors affecting the implementation of PCDC. Staff being older (36), having lower education (38), not understanding residents’ preferences (6), forcing residents to accept unwanted care (6), and negative attitude towards PCDC (32) were identified as barriers to PCDC in both Western and Asian countries, while staff’s rich past work experience (34), high educational background (34), skill in assessing residents’ preferences (51), meet residents’ needs (46), respect for residents (50), receiving positive feedback from residents and perceiving PCDC (49) as important were facilitators. Nursing and care staff in Asian countries faced some barriers not mentioned in Western studies; in particular, nursing staff experienced injuries from people living with dementia and also experienced stigma associated with working in dementia care. The cause of such injuries is not known but the behaviour of those with dementia would most likely result from a combination of factors connected with cognitive impairment and reactions connected with fear. Injuries from residents led to caregivers feeling hurt and helpless, and became a barrier that discouraged them from providing person-centred care (32). Studies of Asian countries found more staffing-related barriers to delivering PCDC compared to Western countries. In Western studies, a unique facilitator was staff empowering the person with dementia to maintain appropriate levels of independence. This was categorized under caregiver factors by the authors of the original study and it implies that this staff skill contributes to staff ability to foster PCDC (43).

#### 3.3.2 People living with dementia and family factors

In both Western and Asian countries, common barriers to PCDC included lack of family support for the work of nursing and care staff, lack of family involvement, family distrust of nurses, unrealistic family expectations, and criticism. Facilitators were when families supported the care staff’s work or noticed the positive effect of PCC practices. The papers reviewed suggested that criticism from families often stemmed from having very high expectations of nursing homes. These unrealistically high expectations could undermine caregivers’ confidence and elevate their psychological stress, impacting their motivation to provide PCDC as ‘nothing is ever good enough (49). Only Western countries reported that family members’ lack of PCDC knowledge was a barrier (43): some family members lacked knowledge of dementia and PCDC, making it difficult for them to accept that the condition of people living with dementia was not improving or was deteriorating. They often believed that the worsening condition was due to the care staff not taking good care of their relatives. Additionally, our study found barriers to delivering PCDC related to individuals with dementia. Staff found the complexities of dementia, including physical impairments and psychological changes, made it hard for them to provide PCDC. In addition, when individuals living with dementia expressed irritability or aggressive behaviour, staff were discouraged from approaching them (48). This hindered PCDC by making it difficult for staff to understand and respond to their needs and preferences. Western studies reported more barriers and facilitators related to individuals with dementia and family factors compared to Asian studies.

#### 3.3.3 Organizational factors

This theme includes factors related to organizational culture, leadership and working arrangements, such as communications, interpersonal relationships, teamwork, education, and training. This factor is less reported in studies of Asian countries than those of Western countries but the subthemes are mostly consistent across both. In Western and Asian studies, the most frequent barriers were inadequate education and training about PCDC for nursing staff (6, 44). In Western studies, a unique barrier identified was the lack of education about dementia for family members. Western studies also reported more issues related to the setup of PCDC activities, such as the need for clear PCDC implementation plans and assistance in prioritizing tasks for staff (56). For Western countries, cohesive team working, leadership support and having an appropriate leadership style were the most common facilitators, while for Asian countries, the most frequent facilitator was PCDC training or education.

#### 3.3.4 Resource factors

Barriers related to lack of resources were common across both Western and Asian countries and included that the physical environment of long-term care facilities did not enable PCDC; lack of staff time; lack of staffing; high turnover of personnel, and wages being too low. Prominent in reports from Western countries were lack of time and staffing to deliver PCDC. Studies of Asian countries also highlighted lack of staffing but, in addition, the poor care environment and low staff wages were prominent barriers (5). Facilitators in Western countries were a warm homely environment and the personalized setting of residents’ rooms but this was also reported in one Asian study (49).

Both Western and Asian studies reported that staff faced heavy workloads, resulting in insufficient time to provide PCDC. Furthermore, studies indicated that high workloads and long hours contributed to employee fatigue, not least as staffing shortages made taking leave a challenge, and training or shift changes sometimes extended working hours (6, 44). These pressures led staff to prioritize rest once basic tasks were done, rather than spending time on PCDC (32, 46). Additionally, high turnover rates in nursing homes undermined team stability, hindering the establishment of relationships between staff and people with dementia, which in turn obstructed the implementation of PCDC (33, 50). Both Western and Asian studies reported low wages and poor benefits for employees, leading staff to feel that their hard work was undervalued, further diminishing their motivation to provide high-quality PCDC (34, 43).

One facilitator related to continuity of staffing which was only reported in Asian studies was that people with dementia saw familiar faces (6). This was seen as contributing to residents’ sense of security, as they experienced greater emotional support through interacting with familiar staff. This facilitator appeared to help staff provide PCDC, as they had the opportunity to develop in-depth understanding of individuals’ histories and interests. This could then be used to evoke memories and facilitate emotional communication. This, in turn impacted on factors associated with the person with dementia, as such relationships enabled individuals to express their needs more comfortably, thereby improving relational understanding and fully embodying the PCDC philosophy.

On the whole, across the studies reviewed, there were shared themes at the higher level, more barriers and facilitators were reported by studies conducted in Western countries than in Asian countries but also both Western and Asian countries had some unique factors.

## 4 Discussion

This study reviewed the literature to establish barriers and facilitators to the implementation of PCDC in long-term care settings in Western and Asian countries. The results were grouped under four high level factors, related to nursing and care staff, people living with dementia and their families, the organization, and resources. Aspects of each are discussed below. We discuss in some depth the possible reasons for several factors and comment on the differences between barriers and facilitators to PCDC in Asian and Western countries.

Among nursing and care staff factors, older age, lower education and lack of knowledge were common barriers to PCDC across both Western and Asian countries, despite the different care contexts. Some studies have shown that the educational level of formal caregivers is positively correlated with the level of PCDC (5). It is possible that this is a more severe barrier in Asian countries. One Chinese study found that 80% of caregivers in nursing homes were illiterate or semi-illiterate, whereas a US study showed that 87% of caregivers had a high school diploma or equivalent (41). The educational level of nurses in Asian countries therefore may limit their understanding, learning and adaptation to the PCDC model (57).

Our review found injuries sustained at work at the hands of people with dementia, and stigma associated with the job were barriers in Asian dementia care but were not reported in Western research. Other researchers have also found that nurses in Asian countries face highly challenging social situations and are more likely to receive physical abuse from people with dementia or their families (56). In addition, in the Asian context, nurses in nursing homes are often perceived as having undignified jobs, resulting in a sense of professional shame (44) and low professional value, which further hampers the motivation of staff to implement PCDC (58). The culture of care in Asian countries focuses mainly on meeting physical needs of residents at the expense of supporting their uniqueness and dignity (5). Care tends to be focused only on basic personal and medical care and fails to support the unique identity of the individual (59). This is not to imply that work with people with dementia is of high status in Western countries, as there are reports of occupational stigma being associated with dementia care work (60), only that this has not been reported as a barrier to PCDC in the studies reviewed.

The Western studies in our review reported unique facilitators in dementia care, emphasizing the role of nurses in empowering individuals with dementia to achieve maximum independence. In PCDC, this empowerment is regarded as essential for enhancing quality of life. Research suggests that encouraging individuals living with dementia to participate in self-care and daily activities, where possible, not only boosts their self-esteem but also improves emotional well-being, potentially slowing cognitive decline (5). Additionally, personalized care plans tailored to individual abilities, can significantly enhance people’s sense of involvement and promote independence (61). This implies that it is imperative that nurses and other care staff prioritize empowerment within their implementation of PCDC to enhance both independence and quality of life for individuals living with dementia.

Most barriers and facilitators linked with people living with dementia and their families were shared across Western and Asian studies. The challenge of implementing PCDC arises from a combination of contextual factors, the nature of cognitive impairment, and the approach taken by staff members. From the perspective of individuals living with dementia, reactions to these factors highlight the difficulties in implementing PCDC. Lack of family involvement and lack of family support are understandable within the different societal contexts of Western and Asian countries. In both Asian and Western cultures, the term “dementia” carries negative connotations (62, 63), with symptoms of dementia leading to significant social and personal stigma. This stigma is particularly pronounced in Asian countries (64) where individuals and their families often feel embarrassed in social contexts (65). Family members may feel guilty for allowing their relatives with dementia to be cared for by others (66, 67). Shame and guilt may cause family members to distance themselves from their relatives with dementia or to place blame for any care issues on the caregivers. This, in turn, can hinder nursing and care staff from providing effective PCDC, as it obstructs access to crucial information about residents’ life histories and personal preferences.

While there were no sub-themes connected with people with dementia and families that were unique to either the Western or Asian context, there was much more detailed and specific reporting of barriers and facilitators in Western studies, while studies in Asian countries were based more on the perceptions of formal caregivers than on practice. This is likely to reflect that PCDC has been embedded in policies, education and guidelines for over two decades in Western countries, whereas Asian countries have less knowledge of PCDC at this stage (19). Similarly, PCDC training, education and support for managers is more lacking in Asian countries (3, 15, 46). While Western countries face some challenges with training resources, they generally provide more comprehensive and specialized educational programs (68). In contrast, Asian countries often experience a significant deficit in specialized training for PCDC, with existing training programs facing several issues, such as scheduling sessions during staff time off, repetitive content, and a disconnect from practical applications (30, 31, 41, 49).

Supportive leadership and teamwork are recognized as important facilitators in both regions. In Western contexts, supportive leadership styles effectively encouraged staff participation in care activities, which is particularly crucial in the emotionally and cognitively complex environment of dementia care (53). Although families play a critical caregiving role within Asian culture (69), only Western studies highlighted the need for educating family members (41). Our findings and the allied literature imply that Asian countries could facilitate better PCDC by fostering family collaboration and involving family members in education of staff.

Lack of resources was widely reported by both Western and Asian studies, with formal caregivers reporting being understaffed and overworked. PCDC is often seen as requiring time to learn and implement, even if this is a misunderstanding (70). The degree of staff and time shortage appears more profound in Asian than in Western long-term care contexts (71). The environment has an impact on the behaviour and health of people with dementia, as well as on care staff, and is a key to good quality of life in long-term care settings (72). Homely environments and opportunities for residents to have individualized rooms were found to be facilitators of PCDC in some studies in Western countries. In contrast, Asian countries have a greater lack of personalised, homely designed environments (5), as well as problems with multiple shared rooms (31), poor lighting, ambient noise, inappropriate use of colour and unclear signage (5). This highlights the benefits to delivering PCDC that are available in more highly-resourced economies. On the other hand, although staff turnover is an issue in both Western and Asian countries, it is reported more frequently in Asian countries. In Western countries, staff groups often include both long-term local staff and young individuals from immigrant backgrounds (73). However, having familiar staff was reported as a positive facilitator of PCDC only in Asian research studies. This suggests that reducing staff churn or turnover in long-term care facilities would be particularly beneficial in enhancing PCDC.

To effectively promote the implementation of PCDC, comprehensive recommendations are proposed across multiple domains. It is suggested that media in both Western and Asian countries have a responsibility to actively disseminate authentic narratives related to dementia care within long-term care facilities, highlighting the efforts and contributions of staff. This would facilitate a more nuanced public understanding of dementia and caregiving roles, and help to reduce stigma associated with dementia and dementia care (74, 75). Educational curricula and continuing professional development need to incorporate modules and courses on PCDC for dementia that foster empathy and understanding among students and nursing and care staff. Information for families and communities that raises awareness about the nature of dementia, particularly in Asian contexts where family responsibilities rooted in Confucian values could be emphasized as a way of encouraging continuing involvement of families. Establishing clear PCDC policies within long-term care facilities would provide a lever to help ensure that staff receive adequate training and support on PCDC. Given the barriers to PCDC revealed through this review that are rooted in lack of resources, it is apparent that further research needs to explore whether better environments can improve resident well-being, so reducing injuries to staff and staff turnover as well as drug costs (76). Arguments around cost-effectiveness of high quality facilities may help to persuade Governments or care companies to invest in better care facilities.

## 5 Strengths and limitations

The strengths of this scoping review are the novelty and relevance of the topic in comparing barriers and facilitators to PCDC in long-term care settings across Western and Asian countries. The review includes a wide range of study types, a comprehensive search of databases, and the inclusion of primary peer-reviewed studies in both English and Chinese. This approach identified articles from 13 countries, contributing to the generalisability of findings. However, it is important to emphasize that these Asian studies are primarily concentrated in China and South Korea, so cannot be generalised across Asia. Our study distills a wide range of barriers and facilitators into four high level themes, offering a systematic framework for understanding both the barriers and facilitators of PCDC. In line with scoping review methodology, studies were not evaluated for quality, which may weaken the strength of our conclusions. In addition, given that Asian studies may be less extensively reported in international journals compared to Western research, it is possible some Asian research was missed. although this was mitigated to some extent by our inclusion of Chinese databases. Furthermore, the exclusive use of English and Chinese databases may have led to the omission of studies in other languages.

## 6 Conclusions

This review found numerous barriers to the implementation of PCDC for individuals with dementia in long-term care facilities across both Western and Asian countries. Barriers are especially pronounced in Asian countries where resources are more limited, implementation is at an earlier stage and nursing and care staff currently have lower educational levels. Additionally, nursing and care staff in Asian countries face unique challenges such as the greater cultural stigma associated with dementia care work, which further impedes the implementation of PCDC. Western studies identified more family-related factors as both barriers and facilitators of PCDC in long-term care facilities; whereas in Asian studies identified organization and resource factors including significant shortages in educational programmes, staffing, and the provision of personalized, home-like environments.

## Data Availability

The original contributions presented in the study are included in the article/**Supplementary Material**. Further inquiries can be directed to the corresponding authors.

## References

[1] Brooker D . Person centred dementia care: making services better. (2006). Available online at: https://www.semanticscholar.org/paper/Person-Centred-Dementia-Care%3A-Making-Services-Brooker/35923261c9700a9a937ae94f93a9f0ab76eea266 (Accessed June 29, 2024).

[2] Sharma T Bamford M Dodman D . Person-centred care: an overview of reviews. Contemp Nurse. (2015) 51:107–20. doi: 10.1080/10376178.2016.1150192 26866531

[3] Kitwood T . Toward a theory of dementia care: ethics and interaction. J Clin Ethics. (1998) 9:23–34. doi: 10.1086/JCE199809103 9598430

[4] World Alzheimer Report 2022 . Life after diagnosis: Navigating treatment, care and support. London, UK: Alzheimer's Disease International (2022).

[5] Zhao Y Liu L Ding Y Chan HYL . Understanding dementia care in care home setting in China: An exploratory qualitative study. Health Soc Care Community. (2021) 29:1511–21. doi: 10.1111/hsc.13213 33118264

[6] Wang J Bian X Wang J . Understanding person-centered dementia care from the perspectives of frontline staff: Challenges, opportunities, and implications for countries with limited long-term care resources. Geriatr Nur (Lond). (2022) 46:39–45. doi: 10.1016/j.gerinurse.2022.04.020 35598581

[7] Wang J . Person-centered care for older adults. In: D Gu Dupre ME , editors. Encyclopedia of gerontology and population aging [Internet]. Springer International Publishing, Cham (2021). p. 3788–94. doi: 10.1007/978-3-030-22009-9_1113

[8] Fazio S Pace D Flinner J Kallmyer B . The fundamentals of person-centered care for individuals with dementia. Gerontologist. (2018) 58:S10–9. doi: 10.1093/geront/gnx122 29361064

[9] Beard JR Officer A de Carvalho IA Sadana R Pot AM Michel JP . The World report on ageing and health: a policy framework for healthy ageing. Lancet Lond Engl. (2016) 387:2145–54. doi: 10.1016/S0140-6736(15)00516-4 PMC4848186 26520231

[10] Røsvik J Brooker D Mjorud M Kirkevold Ø . What is person-centred care in dementia? Clinical reviews into practice: the development of the VIPS practice model. Rev Clin Gerontol. (2013) 23:155–63. doi: 10.1017/S0959259813000014

[11] WHO . Progress report on the united nations decade of healthy ageing, 2021-2023. Geneva, Switzerland: World Health Organization (2023).

[12] Nisbett RE . The geography of thought: How Asians and Westerners think differently … and why Vol. xxiii. . New York, NY, US: Free Press (2003). 263 p. (The geography of thought: How Asians and Westerners think differently … and why).

[13] Drew (PhD) C . Collectivism vs. Individualism: Similarities and Differences(2024). Available online at: https://helpfulprofessor.com/collectivism-vs-individualism/ (Accessed December 2, 2024).

[14] Sorge A Hofstede G . Culture’s consequences: international differences in work-related values. Adm Sci Q. (1983) 28:625. doi: 10.2307/2393017

[15] Mitton C Adair CE McKenzie E Patten SB Waye Perry B . Knowledge transfer and exchange: review and synthesis of the literature. Milbank Q. (2007) 85:729–68. doi: 10.1111/j.1468-0009.2007.00506.x PMC2690353 18070335

[16] Berntsen GR Yaron S Chetty M Canfield C Ako-Egbe L Phan P . Person-centered care (PCC): the people’s perspective. Int J Qual Health Care. (2021) 33:ii23–6. doi: 10.1093/intqhc/mzab052 PMC8633901 34849959

[17] Güney S Karadağ A El-Masri M . Perceptions and experiences of person-centered care among nurses and nurse aides in long term residential care facilities: A systematic review of qualitative studies. Geriatr Nurs N Y N. (2021) 42:816–24. doi: 10.1016/j.gerinurse.2021.04.005 34090225

[18] Kim SK Park M . Effectiveness of person-centered care on people with dementia: a systematic review and meta-analysis. Clin Interv Aging. (2017) 12:381–97. doi: 10.2147/CIA.S117637 PMC5322939 28255234

[19] Lee JY Yang E Lee KH . Experiences of implementing person-centered care for individuals living with dementia among nursing staff within collaborative practices: A meta-synthesis. Int J Nurs Stud. (2023) 138:104426. doi: 10.1016/j.ijnurstu.2022.104426 36584446

[20] Stearns PN . Western civilization in world history. New York, NY: Routledge (2003).

[21] About: List of Asian countries and regions. (2024). Available online at: https://dbpedia.org/page/List_of_sovereign_states_and_dependent_territories_in_Asia (Accessed September 9, 2024).

[22] Arksey H O’Malley L . Scoping studies: towards a methodological framework. Int J Soc Res Methodol. (2005). doi: 10.1080/1364557032000119616

[23] Tricco AC Lillie E Zarin W O’Brien KK Colquhoun H Levac D . PRISMA extension for scoping reviews (PRISMA-scR): checklist and explanation. Ann Intern Med. (2018) 169:467–73. doi: 10.7326/M18-0850 30178033

[24] Peters M Godfrey C Mcinerney P Soares C Khalil H Parker D . Methodology for JBI scoping reviews. Adelaide, Australia: Joanna Briggs Institute (2015) 1–24.

[25] Braun V Clarke V Hayfield N Davey L Jenkinson E . Doing reflexive thematic analysis. In: Bager-Charleson S McBeath A , editors. Supporting research in counselling and psychotherapy : qualitative, quantitative, and mixed methods research [Internet]. Springer International Publishing, Cham (2022). p. 19–38. doi: 10.1007/978-3-031-13942-0_2

[26] Backman A Ahnlund P Lövheim H Edvardsson D . Nursing home managers’ descriptions of multi-level barriers to leading person-centred care: A content analysis. Int J Older People Nurs. (2024) 19:e12581. doi: 10.1111/opn.12581 37859588

[27] Kim D Choi YR Lee YN Chang SO . Nurses’ Shared subjectivity on person-centered care for behavioral and psychological symptoms of dementia in nursing homes. J Nurs Res JNR. (2024) 32:e330. doi: 10.1097/jnr.0000000000000611 38727209

[28] Ehk S Petersson S Khalaf A Nilsson M . Nurses’ experiences of integrating the salutogenic perspective with person-centered care for older people in Swedish nursing home care: an interview-based qualitative study. BMC Geriatr. (2024) 24:262. doi: 10.1186/s12877-024-04831-7 38500060 PMC10946094

[29] Shrestha S Tranvåg O . Dementia care in Nepalese old age homes: Critical challenges as perceived by healthcare professionals. Int J Older People Nurs. (2022) 17:e12449. doi: 10.1111/opn.12449 35139253

[30] Strøm BS Lausund H Rokstad AMM Engedal K Goyal A . Nursing staff's knowledge and attitudes towards dementia in an Indian nursing home: a qualitative interview study. Dement Geriatr Cogn Dis Extra (2021) 11(1):29–37. doi: 10.1159/000514092 PMC7989670 33790938

[31] Park M Jeong H Giap TT . A predictive model of the perceptions of patient-centered care among nurses in long-term care hospitals: A cross-sectional study. Geriatr Nur. (2021) 42(3):687–93. doi: 10.1016/j.gerinurse.2021.02.019 33831715

[32] Kong EH Kim H Kim H . Nursing home staff’s perceptions of barriers and needs in implementing person-centred care for people living with dementia: A qualitative study. J Clin Nurs. (2022) 31:1896–906. doi: 10.1111/jocn.v31.13-14 33624338

[33] Choi J Kim DE Yoon JY . Person-centered care environment associated with care staff outcomes in long-term care facilities. (2021) 29(1):e133. doi: 10.1097/JNR.0000000000000412 PMC7808347 33252502

[34] Chang H Gil C Kim H Bea H . Person-centered care, job stress, and quality of life among long-term care nursing staff. J Nurs Res JNR. (2020) 28:e114. doi: 10.1097/JNR.0000000000000398 32675736

[35] Zhou YP Liu J Li T Wang Y . A qualitative study on the dementia care experience of senior caregivers in elderly care institutions in China. Med Res And Education. (2023) 40:64–70. doi: 10.3969/j.issn.1674-490X.2023.01.010

[36] Zhao YJ Lü XZ Li LY Li C Xia MM Xiao HM . Study on the promoting and hindering factors for implementing person-centered care for dementia patients in long-term care institutions. Chin Nurs Management. (2021) 21:1491–6. doi: 10.3969/j.issn.1672-1756.2021.10.012

[37] Dai Y Zhao J Li S Zhao C Gao Y Johnson CE . Caregivers’ Dementia knowledge and care approach in residential aged care facilities in China. Am J Alzheimers Dis Dementiasr. (2020) 35:153331752093709. doi: 10.1177/1533317520937096 PMC10624015 33089701

[38] Mao P Xiao LD Zhang MX Xie FT Feng H . Investigation on the current situation of person-centered care in dementia care units in nursing institutions in hunan province. J Nurs Science. (2016) 31:1–4. doi: 10.3870/j.issn.1001-4152.2016.21.001

[39] Richter C Fleischer S Langner H Meyer G Balzer K Köpke S . Factors influencing the implementation of person-centred care in nursing homes by practice development champions: a qualitative process evaluation of a cluster-randomised controlled trial (EPCentCare) using Normalization Process Theory. BMC Nurs. (2022) 21:182. doi: 10.1186/s12912-022-00963-6 35804407 PMC9264574

[40] Røen I Kirkevold Ø Testad I Selbæk G Engedal K Bergh S . Person-centered care in Norwegian nursing homes and its relation to organizational factors and staff characteristics: a cross-sectional survey. Int Psychogeriatr. (2018) 30:1279–90. doi: 10.1017/S1041610217002708 PMC6190067 29198221

[41] Lindner H Kihlgren A Pejner MN . Person-centred care in nursing homes during the COVID-19 pandemic: a cross sectional study based on nursing staff and first-line managers’ self-reported outcomes. BMC Nurs. (2023) 21,22:276. doi: 10.1186/s12912-023-01437-z PMC10440872 37605177

[42] Stacpoole M Hockley J Thompsell A Simard J Volicer L . Implementing the Namaste Care Program for residents with advanced dementia: exploring the perceptions of families and staff in UK care homes. Ann Palliat Med. (2017) 6:327–39. doi: 10.21037/apm.2017.06.26 28754045

[43] Hennelly N O’Shea E . A multiple perspective view of personhood in dementia. Ageing Soc. (2022) 42:2103–21. doi: 10.1017/S0144686X20002007

[44] Colomer J De Vries J . Person-centred dementia care: a reality check in two nursing homes in Ireland. Dementia. (2016) 15:1158–70. doi: 10.1177/1471301214556132 25370077

[45] Chu CH Quan AML Gandhi F McGilton KS . Perspectives of substitute decision-makers and staff about person-centred physical activity in long-term care. Health Expect. (2022) 25:2155–65. doi: 10.1111/hex.13381 PMC9615080 34748256

[46] Hunter PV Hadjistavropoulos T Kaasalainen S . A qualitative study of nursing assistants’ awareness of person-centred approaches to dementia care. Ageing Soc. (2016) 36:1211–37. doi: 10.1017/S0144686X15000276

[47] Seah SSL Chenoweth L Brodaty H . Person-centred Australian residential aged care services: how well do actions match the claims? Ageing Soc. (2022) 42:2914–39. doi: 10.1017/S0144686X21000374

[48] Oppert ML O’Keeffe VJ Duong D . Knowledge, facilitators and barriers to the practice of person-centred care in aged care workers: a qualitative study. Geriatr Nur (Lond). (2018) 39:683–8. doi: 10.1016/j.gerinurse.2018.05.004 29859699

[49] Chenoweth L Jeon YH Stein-Parbury J Forbes I Fleming R Cook J . PerCEN trial participant perspectives on the implementation and outcomes of person-centered dementia care and environments. Int Psychogeriatr. (2015) 27:2045–57. doi: 10.1017/S1041610215001350 26307245

[50] Bhattacharyya KK Craft Morgan J Burgess EO . Person-centered care in nursing homes: potential of complementary and alternative approaches and their challenges. J Appl Gerontol. (2022) 41:817–25. doi: 10.1177/07334648211023661 34114482

[51] Kolanowski A Van Haitsma K Penrod J Hill N Yevchak A . Wish we would have known that!” Communication Breakdown Impedes Person-Centered Care. Gerontologist. (2015) 55:S50–60. doi: 10.1093/geront/gnv014 26055781

[52] Doyle PJ Rubinstein RL . Person-centered dementia care and the cultural matrix of othering. Gerontologist. (2014) 54:952–63. doi: 10.1093/geront/gnt081 23921807

[53] Kunz LK Scheibe S Wisse B Boerner K Zemlin C . From dementia mindsets to emotions and behaviors: Predicting person-centered care in care professionals. Dementia. (2022) 21:1618–35. doi: 10.1177/14713012221083392 PMC9234781 35514064

[54] Boumans J Van Boekel L Kools N Scheffelaar A Baan C Luijkx K . How staff characteristics influence residential care facility staff’s attitude toward person-centered care and informal care. BMC Nurs. (2021) 20:217. doi: 10.1186/s12912-021-00743-8 34724935 PMC8559399

[55] Rutten JER Backhaus R Tan F Prins M Roest H Heijkants C . Work environment and person-centred dementia care in nursing homes—A cross-sectional study. J Nurs Manage. (2021) 29:2314–22. doi: 10.1111/jonm.13386 PMC8597031 34053141

[56] Kloos N Drossaert CHC Trompetter HR Bohlmeijer ET Westerhof GJ . Exploring facilitators and barriers to using a person centered care intervention in a nursing home setting. Geriatr Nur (Lond). (2020) 41:730–9. doi: 10.1016/j.gerinurse.2020.04.018 32460962

[57] Sengupta M Harris-Kojetin LD Ejaz FK . A national overview of the training received by certified nursing assistants working in U. S. Nurs homes. Gerontol Geriatr Educ. (2010) 31:201–19. doi: 10.1080/02701960.2010.503122 20730649

[58] Zhu R Hou W Wang L Zhang C Guo X Luo D . Willingness to purchase institutionalised elderly services and influencing factors among Chinese older adults: a nationwide cross-sectional study. BMJ Open. (2024) 14:e082548. doi: 10.1136/bmjopen-2023-082548 PMC10936526 38471688

[59] Yuan Q Yi X Zhang Y Chen Y Qin L Liu H . Study on the current situation and research progress of nursing assistants’ Stress in nursing homes. Chin Nurs Manage. (2015) 15:112–5. doi: 10.3969/j.issn.1672-1756.2015.01.035

[60] Manchha AV Way KA Tann K Thai M . The social construction of stigma in aged-care work: implications for health professionals’ Work intentions. Gerontologist. (2022) 62:994–1005. doi: 10.1093/geront/gnac002 35018434 PMC9372892

[61] Wang Q Xiao X Zhang J Jiang D Wilson A Qian B . The experiences of East Asian dementia caregivers in filial culture: a systematic review and meta-analysis. Front Psychiatry. (2023) 14:1173755. doi: 10.3389/fpsyt.2023.1173755 37151975 PMC10160681

[62] Devenney EM Anh N Nguyen Q Tse NY Kiernan MC Tan RH . A scoping review of the unique landscape and challenges associated with dementia in the Western Pacific region. Lancet Reg Health West Pac. (2024), 50:101192. doi: 10.1016/j.lanwpc.2024.101192 PMC11471059 39399870

[63] Racine L Ford H Johnson L Fowler-Kerry S . An integrative review of Indigenous informal caregiving in the context of dementia care. J Adv Nurs. (2022) 78:895–917. doi: 10.1111/jan.15102 34806198

[64] World Alzheimer Report . Global changes in attitudes to dementia. (2024). Available online at: https://www.alzint.org/resource/world-alzheimer-report-2024/ (Accessed September 26, 2024).

[65] van Corven CTM Bielderman A Wijnen M Leontjevas R Lucassen PLBJ Graff MJL . Empowerment for people living with dementia: An integrative literature review. Int J Nurs Stud. (2021) 124:104098. doi: 10.1016/j.ijnurstu.2021.104098 34706313

[66] Lee KH Lee JY Kim B . Person-centered care in persons living with dementia: A systematic review and meta-analysis. Gerontologist. (2022) 62:e253–64. doi: 10.1093/geront/gnaa207 PMC9019632 33326573

[67] He J Yao Y Jiang L Liu D Jiang M Zhang Y . Analysis of the intentions of home-based pension of the elderly in China and related factors: A nationwide cross-sectional study. Military Nursing. (2024) 41:41–5. doi: 10.3969/j.issn.2097-1826.2024.04.011

[68] Surr CA Gates C Irving D Oyebode J Smith SJ Parveen S . Effective dementia education and training for the health and social care workforce: A systematic review of the literature. Rev Educ Res. (2017) 87:966–1002. doi: 10.3102/0034654317723305 28989194 PMC5613811

[69] Low LF Purwaningrum F . Negative stereotypes, fear and social distance: a systematic review of depictions of dementia in popular culture in the context of stigma. BMC Geriatr. (2020) 20:477. doi: 10.1186/s12877-020-01754-x 33203379 PMC7670593

[70] George ES Kecmanovic M Meade T Kolt GS . Psychological distress among carers and the moderating effects of social support. BMC Psychiatry. (2020) 20:154. doi: 10.1186/s12888-020-02571-7 32252700 PMC7137514

[71] Wang J Wu B Bowers BJ Lepore MJ Ding D McConnell ES . Person-centered dementia care in China: A bilingual literature review. Gerontol Geriatr Med. (2019) 5:2333721419844349. doi: 10.1177/2333721419844349 31192275 PMC6540483

[72] Haunch K Downs M Oyebode J . [amp]]lsquo;Making the most of time during personal care’: nursing home staff experiences of meaningful engagement with residents with advanced dementia. Aging Ment Health. (2023) 27:2346–54. doi: 10.1080/13607863.2023.2177254 36786726

[73] The King’s Fund . The adult social care workforce in A nutshell. (2024). Available online at: https://www.kingsfund.org.uk/insight-and-analysis/data-and-charts/social-care-workforce-nutshell (Accessed December 2, 2024).

[74] Kong D Chen A Zhang J Xiang X Lou WQV Kwok T . Public discourse and sentiment toward dementia on chinese social media: machine learning analysis of weibo posts. J Med Internet Res. (2022) 24:e39805. doi: 10.2196/39805 36053565 PMC9482068

[75] Yang Y Fan S Chen W Wu Y . Broader open data needed in psychiatry: practice from the psychology and behavior investigation of chinese residents. Alpha Psychiatry. (2024) 25:564–5. doi: 10.5152/alphapsychiatry.2024.241804 PMC11443289 39360297

[76] Yao Q Zhang X Wu Y Liu C . Decomposing income-related inequality in health-related quality of life in mainland China: a national cross-sectional study. BMJ Glob Health. (2023) 8:e013350. doi: 10.1136/bmjgh-2023-013350 PMC10689391 38035731

