# Supplementary material for: Barriers and facilitators to implementing person-centred dementia care in long-term care facilities in Western and Asian countries: a scoping review
Source: Front Psychiatry. 2025 Jan 14;15:1523501. doi: 10.3389/fpsyt.2024.1523501 (PMC11772481; doi:10.3389/fpsyt.2024.1523501)
Supplement: Supplementary file 1 [file Table1.docx]

**Table S1. Search terms**

|  | Concept 1 | Concept 2 | Concept 3 |
| --- | --- | --- | --- |
| Key concepts | dementia | person-centered care | Long term care |
| Free text terms /  natural language terms | Dementia* OR alzheimer* | Patient-Centered Care OR person centered care OR Patient centred OR Person centred OR person centered dementia care OR Patient Centered Nursing OR person centered care OR person centred care OR patient centred care OR person centered dementia care OR person centred dementia care OR Patient Centered Nursing | Long-Term Care OR Long term care settings OR nursing home* OR residential home* OR care home* OR assisted living OR residential aged care |
| Controlled vocabulary terms / Subject terms | [mesh]  Dementia OR Alzheimer Disease | [mesh]  Patient-Centered Care | [mesh]  Long-Term Care |

**Table S2. Search trail**

| **Database** | **Search Structure** |
| --- | --- |
| **Pubmed** | (((((((((((Patient-Centered Care[MeSH Terms]) OR ("person centered care"[Title/Abstract])) OR (“Patient centred”[Title/Abstract])) OR (“Person centred”[Title/Abstract])) OR ("person centered dementia care"[Title/Abstract])) OR ("Patient Centered Nursing"[Title/Abstract])) OR ("person centered care"[Title/Abstract])) OR ("person centred care"[Title/Abstract])) OR ("patient centred care"[Title/Abstract])) OR ("person centered dementia care"[Title/Abstract])) OR ("person centred dementia care"[Title/Abstract])) OR ("Patient Centered Nursing"[Title/Abstract])  **AND**  (((((((((((((Perspective*[Title/Abstract]) OR (view*[Title/Abstract])) OR (attitude*[Title/Abstract])) OR (Opinion*[Title/Abstract])) OR (Understanding[Title/Abstract])) OR (experience*[Title/Abstract])) OR (meaning[Title/Abstract])) OR (Belief*[Title/Abstract])) OR (Customs[Title/Abstract])) OR ("cross cultur*"[Title/Abstract])) OR (cultur*[Title/Abstract])) OR (ethnic*[Title/Abstract])) OR (Barrier*****[Title/Abstract])) OR (Facilitator*[Title/Abstract])  **AND**  (((((((Long Term Care[MeSH Terms]) ) OR ("nursing home*"[Title/Abstract])) OR ("residential home*"[Title/Abstract])) OR ("care home*"[Title/Abstract])) OR ("assisted living"[Title/Abstract])) OR ("residential aged care"[Title/Abstract])) OR (longterm care[Title/Abstract])  **AND**  (((Dementia[MeSH Terms]) OR (Alzheimer Disease[MeSH Terms])) OR (Dementia*[Title/Abstract])) OR (alzheimer*[Title/Abstract]) |
| **Web of Science** | AB=(“Patient-Centered Care” OR “person centered care” OR “Patient centred” OR “Person centred” OR “person centered dementia care” OR “Patient Centered Nursing” OR “person centered care” OR “person centred care” OR “patient centred care” OR “person centered dementia care” OR “person centred dementia care” OR “Patient Centered Nursing”)  **AND**  AB=(Perspective* OR view* OR attitude* OR Opinion* OR Understanding OR experience* OR meaning OR Belief* OR Customs OR cross cultur* OR cultur* OR ethnic* OR Barrier* OR Facilitator*)  **AND**  AB=(“Long-Term Care” OR “Long term care” OR “nursing home*” OR “residential home*” OR “care home*” OR “assisted living” OR “residential aged care” OR “longterm care”)  **AND**  AB=(Dementia* OR alzheimer*) |
| **EBSCO—PsycINFO** | AB(“Patient-Centered Care” OR “person centered care” OR “Patient centred” OR “Person centred” OR “person centered dementia care” OR “Patient Centered Nursing” OR “person centered care” OR “person centred care” OR “patient centred care” OR “person centered dementia care” OR “person centred dementia care” OR “Patient Centered Nursing”)  **AND**  AB(Perspective* OR view* OR attitude* OR Opinion* OR Understanding OR experience* OR meaning OR Belief* OR Customs OR cross cultur* OR cultur* OR ethnic* OR Barrier* OR Facilitator*)  **AND**  AB(“Long-Term Care” OR “Long term care” OR “nursing home*” OR “residential home*” OR “care home*” OR “assisted living” OR “residential aged care” OR “Longterm care”)  **AND**  AB(Dementia* OR alzheimer*) |
| **Cumulative Index to Nursing and Allied Health Literature** | AB(“Patient-Centered Care” OR “person centered care” OR “Patient centred” OR “Person centred” OR “person centered dementia care” OR “Patient Centered Nursing” OR “person centered care” OR “person centred care” OR “patient centred care” OR “person centered dementia care” OR “person centred dementia care” OR “Patient Centered Nursing”)  **AND**  AB(Perspective* OR view* OR attitude* OR Opinion* OR Understanding OR experience* OR meaning OR Belief* OR Customs OR cross cultur* OR cultur* OR ethnic* OR Barrier* OR Facilitator*)  **AND**  AB(“Long-Term Care” OR “Long term care” OR “nursing home*” OR “residential home*” OR “care home*” OR “assisted living” OR “residential aged care” OR “Longterm care”)  **AND**  AB(Dementia* OR alzheimer*) |
| **Cochrane Library** | Title abstract keywords(“Patient-Centered Care” OR “person centered care” OR “Patient centred” OR “Person centred” OR “person centered dementia care” OR “Patient Centered Nursing” OR “person centered care” OR “person centred care” OR “patient centred care” OR “person centered dementia care” OR “person centred dementia care” OR “Patient Centered Nursing”)  **AND**  Title abstract keywords(Perspective* OR view* OR attitude* OR Opinion* OR Understanding OR experience* OR meaning OR Belief* OR Customs OR cross cultur* OR cultur* OR ethnic* OR Barrier* OR Facilitator*)  **AND**  Title abstract keywords(“Long-Term Care” OR “Longterm care” OR “nursing home*” OR “residential home*” OR “care home*” OR “assisted living” OR “residential aged care” OR “long term care)  **AND**  Title abstract keywords(Dementia* OR alzheimer*) |
| **EMBASE** | (perspective*:ti,ab,kw OR view*:ti,ab,kw OR attitude*:ti,ab,kw OR opinion*:ti,ab,kw OR understanding:ti,ab,kw OR experience*:ti,ab,kw OR meaning:ti,ab,kw OR belief*:ti,ab,kw OR customs:ti,ab,kw OR 'cross cultur*':ti,ab,kw OR cultur*:ti,ab,kw OR ethnic*:ti,ab,kw OR **barrier*:ti,ab,kw OR facilitator***:ti,ab,kw)  **AND**  ('patient-centered care':ti,ab,kw OR 'patient centred':ti,ab,kw OR 'person centred':ti,ab,kw OR 'person centered care':ti,ab,kw OR 'person centred care':ti,ab,kw OR 'patient centred care':ti,ab,kw OR 'person centered dementia care':ti,ab,kw OR 'person centred dementia care':ti,ab,kw OR 'patient centered nursing':ti,ab,kw)  **AND**  ('long-term care':ti,ab,kw OR 'long term care':ti,ab,kw OR 'nursing home*':ti,ab,kw OR 'residential home*':ti,ab,kw OR 'care home*':ti,ab,kw OR 'assisted living':ti,ab,kw OR 'residential aged care':ti,ab,kw OR 'longterm care':ti,ab,kw)  **AND**  (dementia*:ti,ab,kw OR alzheimer*:ti,ab,kw) |
| **Scopus** | Title abstract keywords(“Patient-Centered Care” OR “person centered care” OR “Patient centred” OR “Person centred” OR “person centered dementia care” OR “Patient Centered Nursing” OR “person centered care” OR “person centred care” OR “patient centred care” OR “person centered dementia care” OR “person centred dementia care” OR “Patient Centered Nursing”)  **AND**  Title abstract keywords(Perspective* OR view* OR attitude* OR Opinion* OR Understanding OR experience* OR meaning OR Belief* OR Customs OR cross cultur* OR cultur* OR ethnic* OR Barrier* OR Facilitator*)  **AND**  Title abstract keywords(**“Long-Term Care” OR “Long term care” OR “nursing home*” OR “residential home*” OR “care home*” OR “assisted living” OR “residential aged care” OR “longterm care**)  **AND**  Title abstract keywords(Dementia* OR alzheimer*) |
| **ProQuest** | abstract(“Patient-Centered Care” OR “person centered care” OR “Patient centred” OR “Person centred” OR “person centered dementia care” OR “Patient Centered Nursing” OR “person centered care” OR “person centred care” OR “patient centred care” OR “person centered dementia care” OR “person centred dementia care” OR “Patient Centered Nursing”)  **AND**  abstract(Perspective* OR view* OR attitude* OR Opinion* OR Understanding OR experience* OR meaning OR Belief* OR Customs OR cross cultur* OR cultur* OR ethnic* OR Barrier* OR Facilitator*)  **AND**  AB=(“Long-Term Care” OR “Longterm care” OR “nursing home*” OR “residential home*” OR “care home*” OR “assisted living” OR “residential aged care”)  **AND**  abstract(Dementia* OR alzheimer*) |
| **Medline** | AB(“Patient-Centered Care” OR “person centered care” OR “Patient centred” OR “Person centred” OR “person centered dementia care” OR “Patient Centered Nursing” OR “person centered care” OR “person centred care” OR “patient centred care” OR “person centered dementia care” OR “person centred dementia care” OR “Patient Centered Nursing”)  **AND**  AB(Perspective* OR view* OR attitude* OR Opinion* OR Understanding OR experience* OR meaning OR Belief* OR Customs OR cross cultur* OR cultur* OR ethnic* OR Barrier* OR Facilitator*)  **AND**  AB(“Long-Term Care” OR “Long term care” OR “nursing home*” OR “residential home*” OR “care home*” OR “assisted living” OR “residential aged care” OR “Longterm care”)  **AND**  AB(Dementia* OR alzheimer*) |
| **CNKI** | 以人为中心 + 以人为本 + 以病人为中心  AND  阿尔茨海默 + 痴呆 + 失智症 |
| **Wanfang** | 主题:(\"以人为中心\" or \"以人为本\" or \"以病人为中心\") and 主题:(\"阿尔茨海默\" or \"痴呆\" or \"失智症\") |
| **CBM** | ("失智症"[常用字段:智能] OR "痴呆"[常用字段:智能] OR "阿尔茨海默"[常用字段:智能])  AND  ("以人为本"[常用字段:智能] OR "以人为中心"[常用字段:智能] OR "以病人为中心"[常用字段:智能]) |
|  |  |
